# Severe Legionella Pneumophila Infection in an Immunocompetent Patient: A Success Story 300 Kilometers Away

**DOI:** 10.7759/cureus.937

**Published:** 2016-12-21

**Authors:** Miguel Jacob, Helena C Ramos, Bruno Morgado

**Affiliations:** 1 Department of Biomedical Sciences and Medicine, University of Algarve; 2 Department of Pneumology, Hospital Centre of Algarve

**Keywords:** outbreak, legionella, legionnaires' disease, pneumonia

## Abstract

The most significant outbreak of Legionella pneumophila, or Legionnaires’ Disease, ever registered in Portugal occurred in 2014, and was considered one of the largest in European history. This relatively rare infection has a dire prognosis if not timely identified and correctly treated, presenting with a high lethality rate. We describe a case of infection by Legionella pneumophila in a previously healthy individual during an outbreak that originated 300 kilometers away from our hospital. The patient presented to the Emergency Department and after an initial assessment, was admitted to the Intensive Care Unit (ICU). He underwent supportive treatment with invasive mechanical ventilation and antibiotic therapy, having been discharged with functional improvement 21 days after admission. During follow-up, the patient presented well without residual clinical or radiological findings. Prompt management following established guidelines allowed for the appropriate treatment and a favorable prognosis. This case serves as a reminder that early management is important, healthy individuals without known risk factors may present with severe infection, and there is the possibility for individual cases of Legionellosis to present far from the outbreak source.

## Introduction

The largest outbreak of Legionnaires’ Disease in Portugal occurred in 2014 and was considered one of the largest ever in Europe, having been described by the World Health Organization as an unusual epidemic and a major public health emergency [1].

Legionnaires' Disease is a rare and important infection that may be associated with high morbidity and mortality (10-30%). Legionella pneumophila has been recognized as a significant cause of community acquired pneumonia (CAP) as well as a cause of both sporadic and epidemic nosocomial pneumonia. Transmission occurs do to inhalation of aerosols from contaminated water sources, and there is no evidence of person-to-person transmission [2]. Cooling towers are the most common associated sources of Legionella outbreaks [3]. Both disease susceptibility and mortality rates are higher in elderly people with associated medical conditions, smokers and patients with a delayed diagnosis and treatment. It can also be associated with alcohol consumption, cancer and immunosuppression [4].

Infection usually results in pneumonia associated with nonspecific findings such as fever, malaise, lethargy, asthenia and myalgia, with no known pathognomonic features [4]. Independent predictors for Legionella pneumonia include elevated body temperature, absence of productive cough, low sodium concentration, high levels of lactate dehydrogenase and C-reactive protein, and thrombocytopenia [5].

We believe the present report is of medical significance due to its epidemiological and public health relevance and the clinical importance of a Legionella outbreak. It serves also as a reminder that healthy individuals without known risk factors may also present with severe infection, early management is important, and there is the possibility that individual cases of Legionellosis may present far from the outbreak source.

Informed consent was obtained from the patient.

## Case presentation

A 46-year-old, non-smoking, male farmer with no known comorbidities presented to the Emergency Department with dyspnea (grade four in the Medical Research Council dyspnea scale), fever, malaise and productive cough.

Physical examination revealed a decrease in vesicular murmur over the left lung field, more evident in the lower two-thirds, and a decrease in the lower third of the right lung field, together with crackles over both hemithoraces. Vital parameters measured on admission are provided in Table 1.

**Table 1:** Vital parameters on admission to the Emergency Department.

Parameter	Value
Respiratory Frequency	22 breaths/min
O_2_ Saturation	86% at ambient air
Pulse	111 beats/min
Arterial Pressure	134/78 mmHg
Temperature	36ºC

Investigations revealed a 94% neutrophilic leukocytosis, a C-reactive protein > 480 mg/L, D-dimers of 4,194 ng/mL, blood urea nitrogen of 28 mg/dL and partial respiratory failure with severe hypoxemia (pO_2_ = 42 mmHg and O_2_ saturation = 72%). A commonly used tool for assessing pneumonia severity assigns points in five areas: confusion, blood urea nitrogen, respiratory rate, blood pressure, and age 65 years or older (CURB-65). Our patient's total CURB-65 score was one (Table 2) [6].

**Table 2:** CURB-65 scoring. BUN: blood urea nitrogen; BP: blood pressure.

Clinical Feature	Points	Points in our case
Confusion	+1	0
BUN > 19 mg/dL (> 7 mmol/L)	+1	1
Respiratory Rate ≥ 30	+1	0
Systolic BP < 90 mmHg or Diastolic BP ≤ 60 mmHg	+1	0
Age ≥ 65	+1	0
Total CURB-65		1

**Figure 1:**
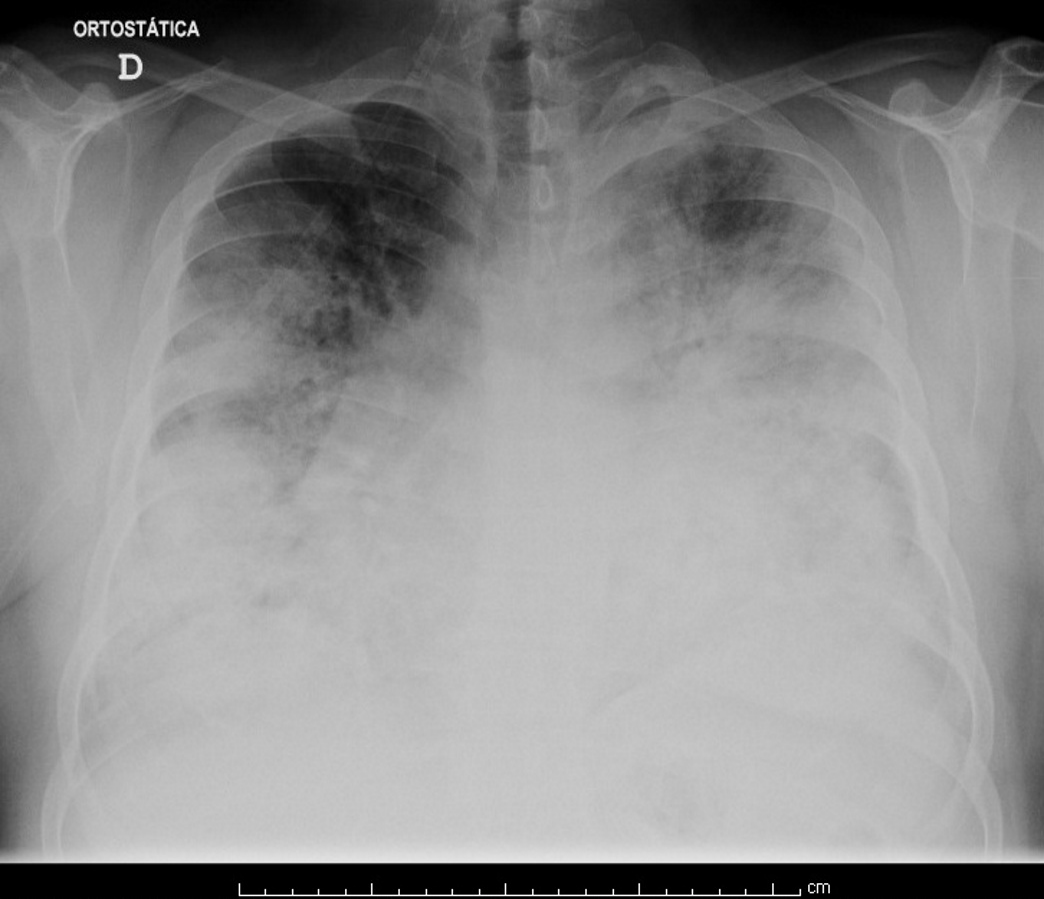
Chest radiography on admission to the Emergency Department. Extensive bilateral infiltrates involving the lower two-thirds of both lung fields can be observed.

Chest radiography showed extensive bilateral infiltrates involving the lower two-thirds of both lung fields, sparing both lung apices (Figure 1).

The patient was admitted to the ICU with the diagnosis of CAP and acute respiratory distress syndrome. Bacteriological investigations of bronchial secretions, blood and urine cultures were negative. He underwent invasive mechanical ventilation and was treated with hydrocortisone and levofloxacin as a single dose in the Emergency Department, which was subsequently replaced with ceftriaxone and azithromycin after admission to the ICU.

On day two he developed oligoanuria while maintaining hemodynamic stability, and treatment with furosemide was started with rapid improvement of renal function and diuresis. Azithromycin was discontinued at day five, ceftriaxone at day 10, and ventilatory weaning occurred at day eight.

**Figure 2:**
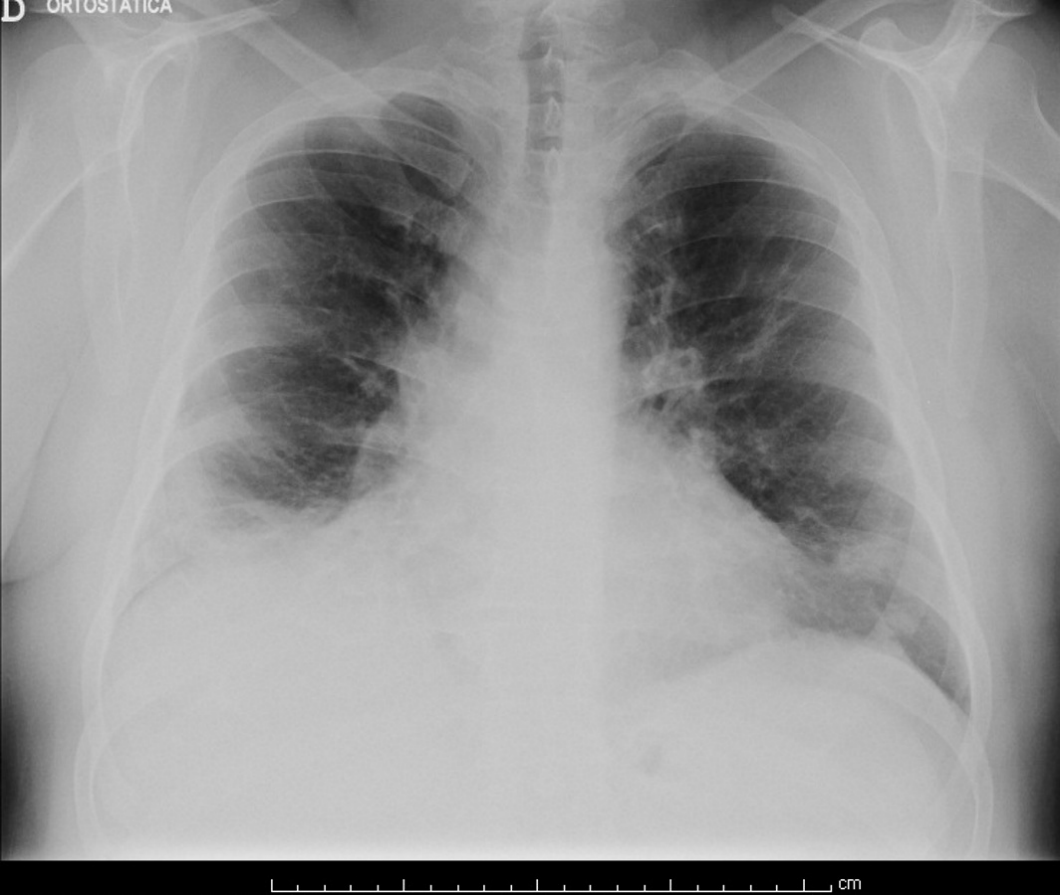
Chest radiography on discharge. An evident decrease in infiltrates can be observed over both lung fields.

During the 17th day after admission, chest radiography was performed which showed significant improvement as only a few residual infiltrates were visible in both lung bases. Serology for Legionella pneumophila and urinary antigen detection tests were positive. Levofloxacin and respiratory kinesiotherapy were started. After clinical, analytical, radiological (Figure 2) and gasometric improvement (Table 3), the patient was discharged 21 days after admission.

**Table 3:** Arterial blood gas findings on discharge.

Parameter	Value at ambient air	Reference interval
pH	7.49	7.35-7.45
pCO_2_	33	35-45
pO_2_	72.4	80-100
HCO_3_^-^	26.1	22-26
O_^2^_ Saturation (%)	96	95-100

**Figure 3:**
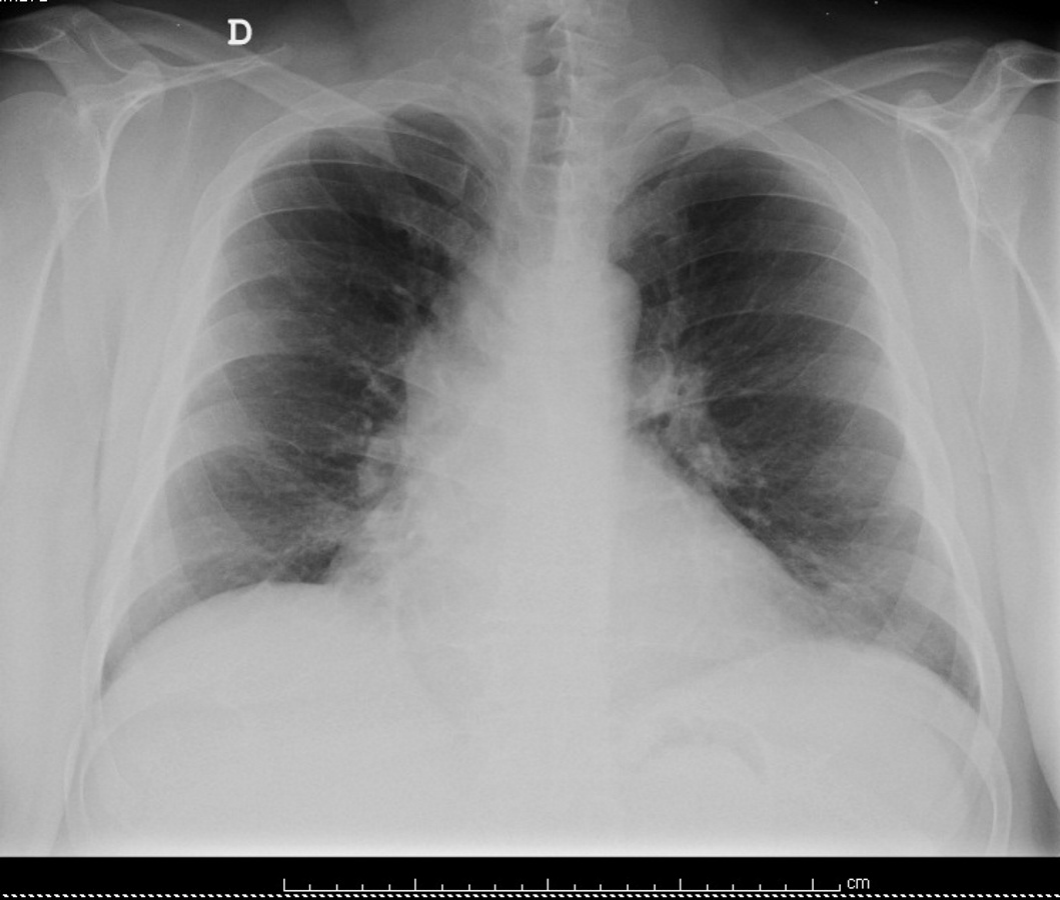
Chest radiography during follow-up. Complete radiological resolution is observed.

At the reassessment appointment, 26 days after discharge, the radiograph revealed signs of complete resolution (Figure 3).

## Discussion

The clinical case described was associated with the above-mentioned outbreak, despite the fact that it presented approximately 300 kilometers away from the infectious center. This fact, associated with the lack of specific symptoms of Legionella infection, no known risk factors and a low CURB-65 score, generated little suspicion for Legionella pneumonia, which could have delayed diagnosis and disease severity assessment.

There are no described pathognomonic radiological findings, although frequent features include progressive infiltrates, which tend to first involve the lower lobes, as was seen on admission [7]. The patient was admitted to the ICU on the same day and placed under invasive mechanical ventilation. Harris et al. stated that late admission to the ICU is associated with a higher mortality rate and that respiratory support should be initiated as soon as possible [8].

The diagnosis of atypical pneumonia is often time consuming. In the present case, given the patient’s clinical findings, the diagnosis of Legionella infection was even more unlikely since, of the independent predictors for Legionella pneumonia, only C-reactive protein was increased in our patient.

Cultures are considered the gold standard test for diagnosis but are frequently not available for timely patient management. Detection of the urinary antigen for Legionella is often used since it is both sensitive and specific, and antigens generally persist in the urine several months after the initial infection [9]. In our case, both urinary antigen and serology for Legionella pneumophila were positive.

The timely use of the urinary antigen detection test for Legionella in combination with appropriate antibiotics, such as azithromycin and levofloxacin, is an approach shown to improve prognosis [10]. Treatment for our patient was started in the Emergency Department with hydrocortisone and levofloxacin, then transitioned to azithromycin with ceftriaxone in the ICU, and later again to levofloxacin.This combination of drugs, although poorly described in the literature for the treatment of Legionnaires' disease, proved to be effective. The patient was consulted one month after discharge and benefited from respiratory kinesiotherapy. Follow-up is important because this condition can be severe enough to exacerbate possible comorbidities, and patients who have been ventilated usually benefit from respiratory rehabilitation [8].

## Conclusions

Legionella pneumophila is an important cause of CAP and nosocomial pneumonia. It is important to recognize that healthy patients without known risk factors may present with severe disease, and epidemiological follow-up, rapid assessment and prompt clinical attitudes, such as ICU admission, are essential for an adequate outcome. Early use of antibiotic therapy on admittance appeared to be a strong prognostic factor in our patient. A complete assessment on admittance is crucial as findings may be misleading, especially when faced with a CURB-65 score of one. Appropriate timing in patient discharge and close follow-up are also important for complete disease resolution.
